# “I was eating more fruits and veggies than I have in years”: a mixed methods evaluation of a fresh food prescription intervention

**DOI:** 10.1186/s13690-021-00657-6

**Published:** 2021-07-23

**Authors:** Cole Heasley, Becca Clayton, Jade Muileboom, Anna Schwanke, Sujani Rathnayake, Abby Richter, Matthew Little

**Affiliations:** 1 Department of Population Medicine, University of Guelph, Guelph, ON Canada; 2 School of Public Health and Social Policy, University of Victoria, Victoria, BC Canada; 3 Guelph Community Health Centre, Guelph, ON Canada; 4 Arrell Food Institute, University of Guelph, Guelph, ON Canada; 5 Food From Thought, University of Guelph, Guelph, ON Canada

**Keywords:** Fresh food prescription, Food security, Community market, Fruits and vegetables

## Abstract

**Background:**

Food insecurity is associated with poor nutritional health outcomes. Prescribing fresh fruits and vegetables in healthcare settings may be an opportunity to link patients with community supports to promote healthy diets and improve food security. This mixed methods study evaluated the impacts of a fresh food prescription pilot program.

**Methods:**

The study took place at two Community Health Centre locations in Guelph, Ontario, Canada. Sixty food insecure patients with ≥1 cardio-metabolic condition or micronutrient deficiency participated in the intervention. Participants were prescribed 12 weekly vouchers to Community Food Markets. We conducted a one-group pre-post mixed-methods evaluation to assess changes in fruit and vegetable intake, self-reported health, food security, and perceived food environments. Surveys were conducted at baseline and follow-up and semi-structured interviews with participants were conducted following the intervention.

**Results:**

Food security and fruit and vegetable consumption improved following the intervention. Food security scores increased by 1.6 points, on average (*p* < 0.001). Consumption of fruits and ‘other’ vegetables (cucumber, celery, cabbage, cauliflower, squashes, and vegetable juice) increased from baseline to follow-up (*p* < 0.05). No changes in self-reported physical or mental health were observed. Qualitative data suggested that the intervention benefited the availability, accessibility, affordability, acceptability, and accommodation of healthy foods for participating households.

**Conclusions:**

Fresh food prescription programs may be a useful model for healthcare providers to improve patients’ food environments, healthy food consumption, and food security.

## Background

Food insecurity, defined as a lack of physical and economic access to sufficient, safe, and nutritious food to meet dietary needs and food preferences [1], is a serious concern in Canadian urban centres. Data from the 2017-2018 Canadian Community Health Survey indicate that approximately 13.9% of households in the city of Guelph (southwestern Ontario, Canada) are food insecure, which is slightly higher than the provincial (13.3%) and national (12.7%) averages [2]. In addition to compromising dietary adequacy, food insecurity is associated with poorer physical, mental, and social health [3, 4]. In particular, food insecure individuals are more likely to suffer from nutrition-related chronic diseases, including hypertension, coronary heart disease, stroke, and diabetes [5–7]. Food insecurity is also burdensome to the healthcare system. Compared to food secure households, annual healthcare costs for marginally, moderately, and severely food insecure households are higher by 23, 49, and 121%, respectively [8]. A primary driver of food insecurity in Guelph region is financial insecurity and poor accessibility of affordable nutritious foods; indeed, a report published in 2018 found that the cost of a nutritious food basket rose 27% between 2009 and 2018, contributing to rising food insecurity [9]. Furthermore, the ongoing COVID-19 public health crisis has exacerbated food insecurity in many Canadian households [10].

Social prescribing programs, in which healthcare providers prescribe (and link patients with) sources of support within their community in lieu of (or in addition to) pharmaceutical prescriptions, are increasing in popularity in the United Kingdom [11, 12] and the United States [13, 14]. Within this field of research, an area for exploration and innovation is food prescription programs. Food prescription programs generally target food insecure patients with diet-related chronic diseases. Such programs often include the provision of vouchers redeemable for healthy foods, access to a nutritionist, and/or access to cooking and nutrition education classes [15–17]. Food prescription programs are intended to improve participants’ food environments by altering one or more of the dimensions of healthy food access: availability (perceived adequacy of supply of healthy foods); accessibility (location and ease of access to healthy foods); affordability (relative cost of healthy foods); acceptability (alignment with food preferences, dietary restrictions, and cultural food practices); and accommodation (meeting the needs of those accessing food) [18]. Overall, there is a need for comprehensive studies to determine the potential of food prescription programs to alleviate food insecurity and improve health while reducing long-term burdens on healthcare systems and reliance on medical interventions.

In 2018, the Guelph Community Health Centre (CHC) (comprised of two physical locations, Downtown and Shelldale, both located in the municipality of Guelph) introduced several initiatives to better link patients with community services [19]. The SEED (capitalized for stylistic purposes; not an acronym), a working project of the Guelph CHC dedicated to addressing food insecurity, leveraged this opportunity to establish a fresh food prescription pilot research project (hereafter FFRx). This research project was grounded in an academic-community collaboration and employed a mixed-methods framework to address two objectives: 1) to assess the impacts of the FFRx program on food security, fruit and vegetable consumption, and self-reported health outcomes; and 2) to examine the impacts of the program on participants’ perceived food environments, including availability, accessibility, affordability, acceptability, and accommodation, through semi-structured interviews.

## Methods

### Recruitment

**Fig. 1:**
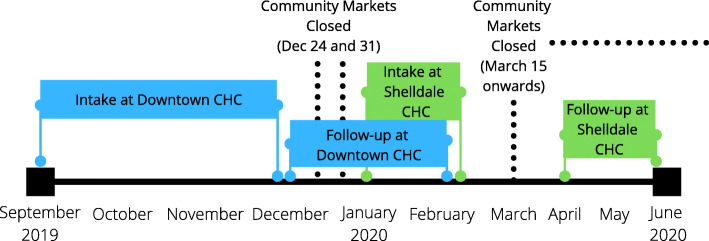
Timeline of research activities during a 12-week fresh food prescription program in Guelph, Ontario, Canada in 2019-2020. Closures from March 15 onwards were due to COVID-19 and public health restrictions

Healthcare practitioners (including nurse practitioners, physicians, dietitians, outreach workers, and social workers) from both the Downtown Guelph and Shelldale CHC locations identified and referred potential participants. Eligible participants included patients who were food insecure and living with a diagnosed cardio-metabolic condition and/or a micronutrient deficiency. Once referred, a representative from the SEED contacted potential participants to offer them a place in the FFRx program. All participants underwent an informed consent process. Recruitment at the Downtown Guelph CHC started late September 2019 and proceeded until December 2019, while recruitment at Shelldale CHC started FFRx in January 2020 and continued for 1 month, ending in February 2020 (Fig. 1). The study protocol was approved by the Research Ethics Board at the University of Guelph (REB #19-06-040).

### The FFRx intervention

The fresh food prescription consisted of food vouchers and an information package. Each participant was provided 12 vouchers to the SEED’s Community Food Markets (hereafter referred to as ‘markets’), valued at ten dollars per person in their household each week up to a maximum of fifty dollars per household per week. The vouchers were redeemable at any of the SEED’s five markets around Guelph, each running one day per week for four hours, including markets at both participating CHCs. The market at the Downtown Guelph CHC was closed for two subsequent weeks during December 2019 since market days fell on statutory holidays. The markets only sold fresh fruits and vegetables, including a variety of seasonal local produce and imported foods.

The vouchers were designed to appear similar to the gift cards already available at the markets to ensure discreetness and minimize stigmatization of participants. Vouchers had an activation date after which they could be redeemed but had no expiry date. If participants did not spend the full voucher amount, market staff recorded any unspent amount and participants could redeem it in subsequent weeks. Participants could pay any outstanding balance with cash or a card if they exceeded the voucher limit. Prices of goods at the market operated on a sliding scale, with the maximum price reflecting approximate grocery store prices and the minimum price 30-50% cheaper. All FFRx participants paid the minimum price on goods by default.

Participants also received a package containing information on the objectives and structure of FFRx, market times and locations, and contact information for the SEED and the study investigators. Information packages also included details on other programs offered by the CHC – for example, the Tasty Tables program (a drop-in healthy cooking class) and the Mindfulness Group (a weekly class on stress, self-care, and group meditation). Participants received ten-dollar coffee shop gift cards as honoraria for both the baseline survey and the follow-up survey and interview.

### Data collection

We conducted a one-group pre-post mixed-methods evaluation of the FFRx intervention. Following informed consent, researchers arranged to meet participants at one of the participating CHC locations for a baseline survey. The survey collected information on demographic details, barriers to cooking, food security, self-reported health outcomes, weekly fruit and vegetable consumption, and involvement in activities and programs in the CHC and wider community. At the end of the 12-week study period, participants were invited to return to repeat the survey and undergo a semi-structured interview to discuss their experiences with FFRx.

The baseline and follow-up survey contained a number of validated questionnaire tools that assessed fruit and vegetable consumption, barriers to cooking, self-reported health, and social capital. Tools included the General Health module of the Canadian Community Health Survey (CCHS) [20] and Harvard Medical School’s Growing Up Today Study questionnaire [21] for self-reported health measures. These survey modules included a checklist of diagnosed health outcomes and five-point Likert scales to record self-reported physical, mental and emotional, and social health status. The CCHS Fruit and Vegetable Consumption module and the Project Eating and Activity over Time (EAT) survey [22] were used for fruit and vegetable consumption and barriers to cooking, respectively. Self-reported consumption of fruits and vegetables was standardized as weekly frequency. The food security assessment tool was based on the CCHS Food Security Module, a 10-item adult scale and 8-item child scale, and results were interpreted using the University of Toronto’s PROOF guidelines [23]. Food security scores were determined as a count of affirmative responses, including “Often true”, “Sometimes true”, or “Yes” to questions such as, “You and other household members worried that food would run out before you were financially able to buy more. Was that often true, sometimes true, or never true in the past four months?” Food security scores were summed, with a higher score representing a higher degree of food insecurity. Reported household food security status was categorized based on whichever score (adult or child) was higher. The module was adapted to reflect the previous 4 months instead of previous year, to capture differences in food security status due to the intervention.

Follow-up semi-structured interviews collected information on overall impressions, voucher usage, perceived benefits and challenges of the program, recommendations for future iterations of the program, and impacts of FFRx on participants’ food environments. Particular emphasis was placed on exploring the impacts of FFRx on food availability, accessibility, affordability, and acceptability and accommodation. For those participants whose enrollment in FFRx was interrupted by COVID-19 closures (described below), interview questions explored the impacts of these closures on self-perceived food security and health. Based on self-reported voucher usage, we identified a subgroup of frequent users, which comprised those participants who redeemed 50% or more of their vouchers.

### Disruption to program due to COVID-19

In mid-March 2020, while the FFRx program was ongoing with Shelldale CHC participants, all markets operated by the SEED were closed due to COVID-19 and subsequent public health restrictions (Fig. 1). As a result, the FFRx program was temporarily disrupted. Following 2 weeks of closures, the SEED shifted to home delivery of fresh produce for FFRx participants. Staff at the SEED selected and delivered food items equivalent to the dollar value of participants’ vouchers every week for the remaining weeks of the program. Following this, participants were invited to take part in the SEED’s Emergency Food Home Delivery program, but their participation in the FFRx research project was concluded. Follow-up with Shelldale participants was conducted over the phone within 6 weeks of participants receiving their final delivery, with the majority within 1 to 3 weeks.

### Data analysis

Descriptive statistics were used to evaluate characteristics of study participants. Pairwise t-tests were used to compare survey data between CHC locations and from baseline and follow-up. All quantitative data analyses were performed in RStudio version 3.6.2. Interview data were analyzed using an inductive-deductive thematic analysis as outlined by Braun and Clark [24]. Qualitative codebooks were created in NVivo 12 Plus (QSR International). Deductive themes (established a priori) were developed by C.H. and M. L. according to the food environment framework introduced by Caspi et al. (2018) and included the impacts of FFRx on five primary dimensions of the food environment: availability, accessibility, affordability, acceptability, and accommodation. Food environment frameworks have been effectively employed to evaluate food and nutrition intervention programs with low-income populations in various settings, and are particularly suited to identify and explore how interventions modulate intersecting determinants of dietary behaviour [25–27]. Additional inductive themes were developed, refined, and confirmed throughout the analysis process. Coding was conducted by C.H. and M.L. working independently, who met to resolve any discrepancies. Codes were grouped into themes and refined, and these themes are presented with quotations (and numerical participant identifiers) to substantiate claims.

## Results

A total of 60 individuals agreed to participate, including 24 participants from the Downtown Guelph CHC and 36 from the Shelldale CHC. The average age of participants at baseline was 47.2 years old (SD ±12.5 years), with a range of 21 to 74 years. The average number of individuals in each household was 2.9 (SD ±1.99; range 1–9), while the average number of children per household was 1.1 (SD ±1.42; range 0–6). A total of 176 household members were captured in the program, although all surveys and interviews were conducted with the primary participant from each household. Survey results are presented in Table 1. Translators were hired by the Shelldale CHC to interpret for participants who spoke non-English languages, including Nepali, Farsi, Dari, Cantonese, Tigrinya, Uzbek, and Vietnamese.

**Table 1:** Descriptive statistics from the baseline and follow-up surveys of a 12-week fresh food prescription program in Guelph, Ontario, Canada in 2019–2020

Characteristic	% (n) or mean (95% confidence interval), baseline survey	% (n) or mean (95% confidence interval), follow-up survey	***p***-value^**a**^ for significance test between baseline and follow-up surveys, if applicable
**Total Participants**	60	36	N/A
**Referring CHC**			
Downtown Guelph CHC	40% (24)	57% (21)	N/A
Shelldale CHC	60% (36)	43% (16)	N/A
**Age Group (years)**			
20 to 34	17% (10)	22% (8)	N/A
35 to 49	32% (19)	30% (11)	N/A
50 to 64	38% (23)	36% (13)	N/A
65+	10% (6)	11% (4)	N/A
Prefer not to answer	3% (2)	0	N/A
**Gender**^**b**^			
Female	77% (46)	71% (25)	N/A
Male	22% (13)	29% (10)	N/A
Prefer not to identify	2% (1)	3% (1)	N/A
**Household Size**			
One	27% (16)	26% (9)	N/A
Two	32% (19)	43% (15)	N/A
Three	8% (5)	3% (1)	N/A
Four	12% (7)	14% (5)	N/A
Five or more	22% (13)	17% (6)	N/A
**Number of Children in Household**			
Zero	53% (32)	58% (21)	N/A
One	13% (8)	11% (4)	N/A
Two	13% (8)	11% (4)	N/A
Three	15% (9)	8% (3)	N/A
Four or more	5% (3)	11% (4)	N/A
**Employment Status**			
Full-time employed	7% (4)	3% (1)	N/A
Part-time employed	15% (9)	8% (3)	N/A
Unable to work because of sickness or disability	38% (23)	39% (14)	N/A
Unemployed	13% (8)	31% (11)	N/A
Retired	15% (9)	8% (3)	N/A
Volunteering	5% (3)	6% (2)	N/A
Looking after family	2% (1)	3% (1)	N/A
Student	3% (2)	3% (1)	N/A
Prefer not to answer	2% (1)	0	N/A
**Receiving Ontario Disability Support Program**			
Yes	52% (31)	64% (23)	N/A
No	48% (29)	36% (13)	N/A
**Estimated Household Annual Income**			
$0 - $9999	15% (9)	N/A	N/A
$10,000 - $19,999	40% (24)	N/A	N/A
$20,000 - $29,000	15% (9)	N/A	N/A
$30,000 - $39,000	5% (3)	N/A	N/A
$40,000 - $49,000	3% (2)	N/A	N/A
$50,000+	3% (2)	N/A	N/A
Do not know / prefer not to answer	18% (11)	N/A	N/A
**Food Security Scores**			
Mean adult food security score	4.1 (3.3–4.9)	2.5 (1.6–3.3)	< 0.001
Mean child food security score	1.9 (0.9–2.9)	0.93 (0.1–0.9)	0.01
**Food Security Status**			
Food secure	3% (2)	25% (9)	N/A
Marginally food insecure	10% (6)	19% (7)	N/A
Moderately food insecure	55% (33)	36% (13)	N/A
Severely food insecure	32% (19)	17% (6)	N/A
Prefer not to answer	0	3% (1)	N/A
**Health outcomes**			
Depression	63% (38)	58% (21)	N/A
Anxiety	57% (34)	50% (18)	N/A
Arthritis	47% (28)	36% (13)	N/A
Iron deficiency anemia	37% (22)	33% (12)	N/A
Hypertension	35% (21)	33% (12)	N/A
Vit D deficiency	31% (19)	22% (8)	N/A
Vit B12 deficiency	28% (17)	19% (7)	N/A
Migraines	28% (17)	42% (15)	N/A
High cholesterol, triglycerides, or lipids	25% (15)	22% (7)	N/A
Diabetes	25% (15)	25% (9)	N/A
Type 1	2% (1)	3% (1)	
Type 2	13% (8)	6% (2)	
Unsure of Type	10% (6)	17% (6)	
Asthma	22% (13)	25% (9)	N/A
Stress fracture	20% (12)	8% (3)	N/A
Hypothyroidism	18% (11)	6% (2)	N/A
Concussions or head injuries	17% (10)	25% (9)	N/A
**Self-reported physical health**			
Mean physical health score	2.3 (1.8–2.8)	2.8 (2.4–3.2)	0.13
Poor	27% (16)	36% (13)	N/A
Unsatisfactory	15% (9)	17% (6)	N/A
Fair	28% (17)	25% (9)	N/A
Good	27% (16)	19% (7)	N/A
Excellent	3% (2)	0	N/A
Prefer not to answer	0	2% (1)	N/A
**Self-reported mental health**			
Mean mental health score	2.4 (1.9–2.8)	2.3 (1.8–2.7)	0.67
Poor	22% (13)	25% (9)	N/A
Unsatisfactory	15% (9)	19% (7)	N/A
Fair	30% (18)	22% (8)	N/A
Good	23% (14)	22% (8)	N/A
Excellent	7% (4)	11% (4)	N/A
Prefer not to answer	3% (2)	0	N/A
**Self-reported social relationships**			
Mean social relationships score	1.7 (1.2–2.2)	1.9 (1.5–2.4)	0.41
Poor	17% (10)	14% (5)	N/A
Unsatisfactory	8% (5)	8% (3)	N/A
Fair	17% (10)	31% (11)	N/A
Good	42% (25)	39% (14)	N/A
Excellent	17% (10)	6% (2)	N/A
Prefer not to answer	0	3% (1)	N/A
**Mean Weekly Frequency of Consumption of Fruits and Vegetables**^**c**^			
Juice	2.4 (0.98–3.8)	2.8 (1.2–4.4)	0.68
Fruit	4.7 (2.3–7.0)	8.5 (5.6–11.5)	0.05
Dark green vegetables	5.5 (3.1–7.9)	5.2 (3.7–6.8)	0.82
Orange vegetables	3.1 (0.74–5.5)	4.2 (2.9–5.6)	0.46
Potatoes	3.2 (1.2–5.2)	2.7 (1.9–3.6)	0.55
Other vegetables	3.5 (2.2–4.7)	5.2 (3.6–6.9)	0.02

^a^*p*-values reflect pairwise t-test for differences in means between baseline and follow up

^b^Responses to gender were open ended (not selected from a checklist)

^c^Dark green vegetables include broccoli, green beans, peas, green peppers, and dark leafy greens like romaine lettuce and spinach. Orange vegetables include carrots, orange bell peppers, sweet potatoes, pumpkin, and squash. Other vegetables include cucumber, celery, corn, cabbage, and vegetable juice

After completing FFRx, 36 out of the 60 participants responded for follow-up surveys and 37 completed a follow-up semi-structured interview. Thirty-four of the interviews were audio recorded, and notes were taken for the other three participants according to participant preference. One participant conducted the semi-structured interview but not the follow-up survey. The survey therefore had a follow-up rate of 83.3% (20/24) with participants from the Downtown Guelph CHC and 44.4% (16/36) with participants from the Shelldale CHC (overall follow-up rate 60%). The overall follow-up rate for interviews was 61.7% (37/60). Retained participants were not notably different from participants lost to follow-up in regard to any outcomes evaluated by baseline surveys (e.g., food security, self-reported health, and fruit and vegetable consumption), alleviating concerns of bias due to loss to follow-up.

Overall, FFRx was viewed positively by participants. During interviews, when asked for comments on their experience in the program, one participant responded, “The program’s fantastic […] I know it’s only for a short amount of time, but it’s definitely appreciated, I guarantee it. I don’t think one person that walks through that door to get vegetables would say they don’t appreciate it” (601). Another participant stated, “My experiences were [ ….] largely fantastic. Given all of my disabilities, everybody was really accommodating and really helpful. And it gave me choices and options that I don’t normally have, and I really appreciated that” (606). Many expressed an interest in the continuation of the program, with one participant stating, “It’s very good. And if that could continue forever, it would be lovely” (1301).

### Impacts of FFRx on food security

During the initial survey, most participants were moderately (55%) or severely (32%) food insecure. The initial average adult food security score and the average change in score were not significantly different between the Downtown Guelph and Shelldale CHC locations (*p* = 0.21 and *p* = 0.14, respectively). For those participants who completed the follow-up survey, we compared mean food security scores at baseline and follow-up (Table 2). For households with a baseline status of marginal, moderate, and severe food insecurity, average reductions of 50, 32, and 47% in adult food security scores were recorded, respectively.

**Table 2:** Food security scores for participants at baseline and follow-up of a 12-week fresh food prescription program in Guelph, Ontario, Canada in 2019–2020

Characteristic	Baseline mean (95% CI) or n (%)	Follow-up mean (95% CI) or n (%)	*p*-value^a^
Food security score (all participants)^b,c^			
Adult score (*n* = 35)	4.1 (3.3–4.9)	2.5 (1.6–3.3)	< 0.001
Child score (*n* = 14)	1.9 (0.92–2.9)	0.93 (0.23–1.6)	0.01
Food security score (Downtown Guelph CHC participants)			
Adult score (*n* = 19)	4.5 (3.4–5.6)	2.5 (1.2–3.7)	< 0.001
Child score (*n* = 8)	2.4 (0.85–4.0)	1.3 (0.21–2.0)	0.05
Food security score (Shelldale CHC participants)			
Adult score (*n* = 16)	3.5 (2.3–4.7)	2.5 (1.2–3.8)	0.14
Child score (*n* = 6)	1.2 (−0.060–2.4)	0.50 (−1.1–2.3)	0.10
Food security score (frequent users^d^)			
Adult score (*n* = 15)	4.1 (2.7–5.5)	1.7 (0.4–3.0)	< 0.001
Child score (*n* = 8)	2.1 (0.1–4.1)	1.0 (0.1–1.9)	0.20
Food security category (*n* = 35)			
Food secure	0 (0%)	9 (25.7%)	
Marginally food insecure	4 (11.4%)	7 (20.0%)	
Moderately food insecure	20 (57.1%)	13 (37.1%)	
Severely food insecure	11 (31.4%)	6 (17.1%)	
Food security score by category at baseline			
Marginally food insecure at baseline (*n* = 4)	1 (1–1)	0.5 (−0.42–1.4)	0.18
Moderately food insecure at baseline (*n* = 20)	3.3 (2.7–3.8)	2.2 (1.0–3.4)	0.07
Severely food insecure at baseline (*n* = 11)	7.0 (6.5–7.5)	3.7 (2.3–5.2)	< 0.001

^a^*p*-values reflect pairwise t-test for differences in means between baseline and follow up

^b^Only includes those participants who responded to follow-up surveys; one participant from Downtown CHC preferred not to answer these questions

^c^Note that a lower food security score indicates a higher level of food security

^d^Includes only those participants that reported using ≥50%of their vouchers

At follow-up, 26 respondents improved their adult food security scores (74%), six households had poorer scores (17%), and three (8.6%) had no change compared to baseline scores. The positive impact of FFRx on household food security was confirmed by participants in the semi-structured interviews. As stated by one participant, “… [FFRx] definitely helped me plan some of my meals and just have nutritious meals through the week, so I found it very good” (301). The six participants for whom household food security worsened during the study claimed in interviews that market closures (due to holidays or the COVID-19 pandemic) and issues with the home delivery program reduced the program accessibility, as discussed in further detail below.

### Impacts of FFRx on self-reported health and fruit and vegetable consumption

No significant changes in self-reported physical or mental health were seen from baseline to follow-up (Table 1), even when examining only those participants who were categorized as frequent users (those who used ≥50% of their vouchers). However, a few participants expressed the perceived beneficial physical health impacts of FFRx in the interviews, and particularly the perceived usefulness of the intervention for improving control of cardiometabolic health. As expressed by one participant “… [participating in FFRx] meant that for those past several months, I didn’t have a diet based on carbohydrates, I could eat a diet that could have a lot more fruits and vegetables and proteins, which is so good for me when I’m [ …] trying to get some diabetes under control” (301).

Downtown Guelph CHC participants and frequent users reported a marginal improvement in social health over the duration of FFRx (+ 0.5 points; *p* = 0.08 among both sub-groups). This improvement may be due to the social environment of food markets, which provided an opportunity for participants to connect with other customers and staff. As expressed by one participant, “Because I have limited use of my hands, [market staff] help me with getting the vegetables that I choose and making sure that I was able to take my time, and chat with them about you know, what I was choosing, and what was going on, and how to use them. It was in general just a really good social thing for me, in addition to bumping up my nutrition” (1206). Shelldale CHC participants did not experience the same benefits to social health and reported an average decline in their social health (− 1.1 points; *p* = 0.01). However, this may be attributed to the exceptional impacts of the COVID-19 pandemic, which was a considerable disruption to markets and individuals’ personal lives during the intervention with Shelldale CHC participants. Market closures and the shift to home food delivery limited the social dimensions of FFRx. As described by one participant, “I [ …] prefer shopping at the market rather than home delivery. Because that way, you get out of the house, talk to people …” (1207).

**Fig. 2:**
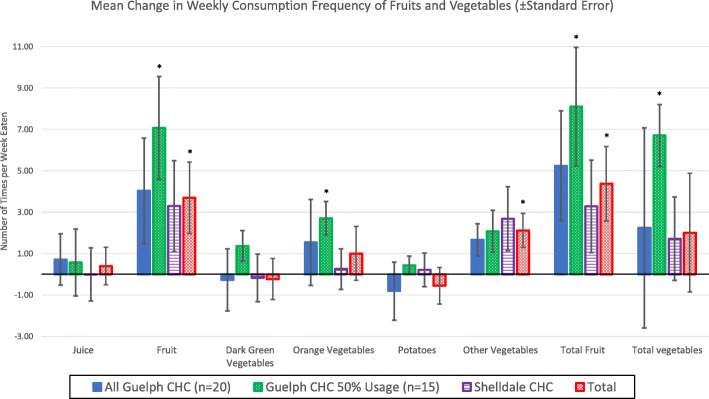
Mean weekly change in frequency of consumption of fruit and vegetables (± standard error) during a 12-week fresh food prescription program at two locations in Guelph, Ontario, Canada in 2019–2020. Total fruit is a sum of juice and fruit consumption frequencies and total vegetables a sum of dark green vegetables, orange vegetables, potatoes, and other vegetables consumption frequencies. *p-value ≤0.05 for comparison of fruit and vegetable consumption at baseline and follow-up

Overall, self-reported frequency of fruit and ‘other’ vegetable (e.g., cucumber, celery, corn, cabbage, and vegetable juice) consumption was higher at follow-up than baseline (Table 3). Notably, frequent users reported a mean increase in frequency of fruit and total vegetable consumption of about once more per day. This may be due to the low mean frequency of consumption among these participants at baseline, suggesting that those individuals who consumed fewer fruits and vegetables prior to enrollment in the program were more likely to use the vouchers. Findings also indicated that those participants who used the program more frequently experienced significant improvements in their total fruit and vegetable consumption (Fig. 2).

**Table 3:** Mean weekly consumption frequency of fruits and vegetables at baseline and follow-up during a 12-week fresh food prescription program in Guelph, Ontario, Canada in 2019–2020

Food category	All participants (*n* = 36)		Downtown Guelph CHC participants (*n* = 20)		Frequent users^†^ (*n* = 15)		Shelldale CHC participants (*n* = 16)	
	Baseline	Follow-up	Baseline	Follow-up	Baseline	Follow-up	Baseline	Follow-up
Juice	2.40	2.80	2.81	3.53	2.69	2.96	1.89	1.88
Fruit	4.69	8.53**	5.15	9.42	3.97	9.18**	4.14	7.43
Dark green vegetables^a^	5.48	5.25	4.94	4.66	4.47	5.03	6.16	5.97
Orange vegetables^b^	3.12	4.25	3.10	4.64	2.15	3.74**	3.15	3.70
Potatoes	3.22	2.74	3.49	2.67	2.45	2.63	2.88	2.83
Other vegetables^c^	3.47	5.23**	1.72	3.35*	3.90	5.83*	5.57	7.74
Total fruit^d^	6.96	11.33**	7.70	12.94*	6.53	12.14**	6.03	9.31
Total vegetables^e^	11.78	14.34	9.59	12.65	5.47	11.74**	14.52	16.47

^†^Includes only those participants that reported using ≥50% of their vouchers; excludes participants from Shelldale CHC due to COVID-19 interruption and shift to food box delivery service

*p-values ≤0.10 in pairwise t-test between baseline and follow-up

**p-values ≤0.05 in pairwise t-test between baseline and follow-up

^a^Dark green vegetables include broccoli, green beans, peas, green peppers, and dark leafy greens like romaine lettuce and spinach

^b^Orange vegetables include carrots, orange bell peppers, sweet potatoes, pumpkin, and squash

^c^Other vegetables include cucumber, celery, corn, cabbage, and vegetable juice

^d^Total fruit is the sum of fruit and juice

^e^Total vegetables is the sum of dark green vegetables, orange vegetables, and other vegetables

### Impacts of FFRx on dimensions of the food environment

We assessed survey data and interview transcripts using an inductive-deductive open coding approach to determine the impacts of FFRx on participants’ perceived food environments across the five dimensions: availability, affordability, accessibility, accommodation, and acceptability.

#### Fruit and vegetable availability, consumption, and dietary diversity

While consumption of fruits and vegetables did not increase universally across locations and subgroups, qualitative data confirmed that participants experienced increased availability and consumption of fresh, high-quality fruits and vegetables due to FFRx. As stated by one participant, “I was eating more fruits and veggies than I have in years” (1304). Another participant claimed that the program allowed them to access fresh food more consistently, stating: “We’re getting access to vegetables [ …] most of the month, whereas before we really only get access for the first week of the month, because [of] my [Ontario Disability Support Program schedule] [ …], and can only buy so much that doesn’t last” (501). Further, several participants mentioned that the program increased dietary diversity by exposing them to novel fresh foods, with potential health benefits. For example, one participant stated, “It’s made me want to experiment and try out new things. And it’s also given me access to things I didn’t normally look for or know about. Like using turmeric and ginger for my inflammation” (1002).

Participation in FFRx had impacts on food availability beyond the program participants. Several participants noted they shared food purchased at the markets with people in their family and/or neighbourhoods. As described by one participant, “So, I share around with everybody who might need it, and where I live, everybody needs it. So, yeah, I try to be nice. ‘Cause it was nice to get it, you know what I mean?... pass it on” (903). Another participant donated food to other services at the CHC, explaining, “A lot of times I will buy extra [fruit] and donate it … over there [*to other CHC facilities*] so, that’s helped, you know, I like to be able to help them as well, since someone’s helping me” (802). When the FFRx adopted home delivery due to the COVID-19 pandemic, participants often shared food with family and neighbours when they received too much of certain foods, or foods that they did not like or could not eat.

#### Affordability of healthy foods

Many participants found that the vouchers and market structure increased the affordability of food. As stated by one participant, “I don’t have enough budget to buy vegetables and fruit, and in this program, I have enough vegetables and fruit for the food for my family every week” (1105). Participants receiving support from the Ontario Disability Support Program (ODSP) and other monthly income support programs (e.g., Employment Insurance) reported that the vouchers supported them to afford fresh fruits and vegetables throughout the entire month, when normally they are only able to afford healthy foods immediately following income support payments. The sliding price scale at the markets also elevated the purchasing power of the vouchers, as prices were often less expensive than at grocery stores. As described by one participant, “Twenty dollars a week for my family made a phenomenal difference with the sliding scale because twenty dollars a week at [a large grocery retailer] is nothing. Twenty dollars a week here, that’s a full week’s groceries of fresh food!” (303). While participants indicated that a few items (e.g., bananas) were more expensive at the markets in comparison grocery stores, they generally perceived foods to be competitively priced, especially since they were regarded as “fresher” than those available at the grocery stores.

The weekly voucher value of ten dollars per person per household was perceived as adequate by most participants. Indeed, many participants found they spent less than the full amount of their weekly vouchers. One participant told us, “It was easy to budget within [the voucher amount] and use up any excess the next time [ …] It was pretty easy to juggle that around. And if I wanted more than the ten dollars one week, I would pay cash” (1206). Conversely, a few participants found it challenging to stay within the voucher limit each week. All participants appreciated the ability to carry over unspent balance from weekly vouchers. As stated by one participant, “… if you don’t use the full amount, it still goes over, and they write the remaining balance on it, which is nice. And they don’t expire” (908). Many participants reported missing some weeks but used multiple vouchers in subsequent weeks. As stated by one participant, “It was really good to be able to carry one over, ‘cause like I said I would have missed out on a lot of weeks, ‘cause probably for about half of the weeks I had to carry another one over” (801). Some participants purchased less than the value of their vouchers to limit food spoilage. As explained by one participant, “… there were many weeks where I got less than twenty dollars because I didn’t want what I was taking home to spoil” (303). Allowing participants to carry over the unspent balance therefore provided participants with flexibility to plan their food purchases in advance and make strategic decisions about food purchases that aligned with household consumption habits.

#### Accessibility: location and timing of markets

The FFRx vouchers were redeemable at any of five markets in Guelph, each running one day per week for four hours. Study participants generally found the markets to be accessible. A benefit of FFRx was that two of the weekly markets were located within the CHCs, allowing participants to easily access CHC services and markets simultaneously. For many participants, the closest market was within walking distance from their homes, which benefited those participants for whom transportation was a challenge. As stated by one participant, “the important thing is the location, so it was convenient for us … it’s walkable from my house, and we don’t have [to take] transportation” (1106).

Some participants were grateful for the multiple markets that occurred on different days. Participants appreciated that that if they missed a market day at their ‘usual’ location, they could access one of the additional markets on a subsequent day. However, others were unable to attend other markets due to transportation and mobility barriers and were thus constrained by the limited hours of the closest weekly markets. One participant noted “the one thing is it only happens one day a week … so if I miss that then I wouldn’t be able to come,” (801) indicating that expanded market days and/or hours would be appreciated.

Participants raised several additional accessibility challenges. Specifically, poor mental health and chronic pain prevented some participants from attending markets. Several participants also had difficulty remembering the market days and hours, although they found it beneficial when reminded by CHC staff and healthcare providers. For some participants who spoke limited English, language barriers were cited as an initial challenge to navigate the program and market environments, although most participants were able to overcome such barriers as they became familiar with the program. As reported by one participant, “… you just need to practice a couple of times to get used to it” (1207). Market closures during the December 2019 holidays posed an accessibility issue for participants. When asked how often they attended the markets, one participant reported, “well because of the Christmas holidays and my anxiety, not as often as I would like. But, yeah, I’ve been trying” (1002). In September 2019, the Downtown Guelph CHC market was moved outside and a block away from the Downtown Guelph CHC for several weeks, which created challenges for participants with mobility impairments. For those participants with physical disabilities, poor weather exacerbated mobility issues. Participants with visual impairments also expressed accessibility challenges at markets. Finally, in follow-up interviews, several participants reported using fewer than half of their vouchers. These participants cited numerous barriers that preventing them from taking full advantage of the program, including challenges remembering the market times, mental health episodes, physical health and mobility, weather, inconvenient locations of markets, and the holiday closures.

#### Acceptability and accommodation: food preferences and the market environment

Overall, the produce available at markets was acceptable to participants. Fruits and vegetables were considered fresh and high-quality. One participant mentioned, “Oh, I really enjoyed it because everything is so clear and fresh, and everything looks amazing. I mean, the cauliflower is white as white can be. You know, and the carrots are a nice vibrant red. You know, nothing’s old, it’s all fresh” (601). The markets also provided produce that was acceptable for participants with dietary restrictions – for example, those who had difficultly chewing and those with food allergies. While some participants described their purchasing choices being limited by their “picky children”, most parents used the program to obtain foods that met the preferences of all family members. Some participants requested ingredients for preparing culturally appropriate dishes. For example, one participant told us, “the SEED [ …] [should] look into some culturally appropriate choices of vegetables, because that will really encourage more folks to eat more healthily, things like mustard greens and callaloo, and those pretty unique vegetables are not necessarily expensive but they’re very, very valuable to people from the immigrant community who don’t know where to access them here or aren’t even aware that it’s possible to find them in Canada, even after year [s] …” (1202).

Most participants experienced a welcoming environment at the markets. The social atmosphere of the markets was a motivator for some to attend the markets regularly, as they enjoyed positive interactions with staff and other shoppers. Since the markets were smaller and quieter than grocery stores, they had a “small community feeling” that made some participants feel more relaxed than shopping at grocery stores as “[the market] felt friendlier, it felt homier” (1202) and was welcoming to parents with children. For some participants, FFRx was a gateway to other CHC programming; indeed, participants reported increased involvement in other CHC services (e.g., the Tasty Tables program and the Mindfulness Group) during the follow-up surveys.

Initially, many participants at the Downtown Guelph CHC felt embarrassed or concerned that they would face judgement and/or stigma at the markets. However, these feelings did not prevent any participants from attending. Such concerns were alleviated by the discreetness of voucher cards and positive interactions with market staff. As stated by one participant, “I felt like it was very discreet, having the card and nobody questioned that, so I found other people were very accepting of my situation and the fact that I’m part of this program” (302). Participants appreciated market staff who were consistently “helpful”, “kind”, “friendly”, and who went out of their way to help shoppers at the market. Staff welcomed participants and engaged with them about produce selection and preparation, which contributed to a positive market experience. Participants socialized with market staff frequently; as one participant stated, “There was times that we’d be done for a good five, ten minutes, and we’d still be just standing there talking” (1303).

Some participants expressed initial concerns about language barriers, and some were initially confused about whether the market would simply subsidize purchases (instead of redeeming dollar vouchers) and if they would have the freedom to select their own produce. These concerns were alleviated when the program was further explained by their referring healthcare practitioner, upon receiving the voucher cards, or once they attended the market. A participant described “when [healthcare practitioner] sent me, I say we don’t know … maybe that I don’t use. But when I went and I see that I can choose the things, I say ‘Oh, that’s perfect’” (1105).

### COVID-19 and transition to home delivery program

Due to the impacts of the COVID-19 pandemic, all markets closed for two weeks in mid-March 2020. During this time, while the program was completed with the Downtown Guelph CHC group, participants with the Shelldale CHC were unable to redeem vouchers. After these two weeks, FFRx participants received weekly home deliveries of fresh produce equal to the value of their vouchers for the remainder of their 12-week enrollment in FFRx. Afterwards, they were invited to enroll in the Emergency Food Distribution Program, a separate program facilitated by the SEED. The food deliveries during this time were appreciated by participants. As stated by one participant, “I said ‘Oh my God, comes from Heaven’ … so it’s like that, I was very excited, it’s like when you receive a parcel, and somebody sends you a gift!” (1408). This program improved participants’ feelings of food security and provided healthier options than what participants were getting at the grocery stores.

While all participants were understanding of the constraints posed by the pandemic, participants did not appreciate that the contents of delivery boxes were preselected, citing the lack of choice as a detriment to the effectiveness of the program. Participants felt their lack of choice led to limited sensitivity towards participants’ dietary restrictions, inappropriate portion sizes, and poor alignment with participants’ diverse cultural preferences and cooking habits. Some participants were unfamiliar with certain vegetables in the delivery boxes, and thus were unable to use them. While some participants enjoyed the surprise of what they would receive in the box or discovered they enjoyed new foods, many participants stated they shared some of their uneaten or undesirable food with family, friends, or neighbours so as not to waste food.

## Discussion

This study undertook a mixed-methods evaluation of a food prescription program incorporating both quantitative measures and qualitative (semi-structured interview) evidence. To our knowledge, FFRx is the first academic-community partnership to initiate and evaluate a food prescription program in Canada. The organizational structure of the program was unique, in that the participating Community Health Centre had the capacity to both issue and honor the vouchers at their internal produce markets, unlike other fresh food prescription programs that relied on external partners for food distribution, such as farmers’ markets and supermarkets [15, 28–30]. Our findings suggest that food prescription programs can effectively link participants with community supports to address food insecurity and improve nutritional health, potentially reducing healthcare burdens and reliance on pharmaceutical and therapeutic interventions. Over its 12-week duration, FFRx improved food security and increased self-reported intake of fruits and some vegetables among participants. Some participants reported parallel improvements in self-reported and perceived health. Our study also showed that FFRx altered the food environments of participants by improving the availability, affordability, accessibility, acceptability, and accommodation of healthy foods. Our evaluation also identified barriers to the program (such as market closures) and pathways to program utilization (such as the beneficial social environment of markets). Findings align with previous program evaluations from the United States and the United Kingdom that have shown food prescription models to improve food security and health outcomes. There remains a necessity for additional rigorous evidence on the impacts of food prescriptions, as research on these interventions frequently suffers from small sample sizes, lack of a control group, and short duration (< 6 months) [29, 31].

Research findings from our study correspond with previous fresh food prescription programs that have been shown to improve food security for individuals and households [16, 32, 33]. A similar program in Georgia, USA found a 33% decrease in participants reporting they “often cut the size of meals or skipped meals due to financial constraints” [33]. An evaluation of the same program in pediatric patients across the USA found that 72% of participating households improved their food security scores following administration of food prescriptions [32]. Similarly, a prescription program in Harris County, Texas, USA recorded a 94% decrease in the prevalence of food insecurity amongst their participants over a six-month intervention [16]. Our study showed that FFRx had similar potential to improve food security, with 74% of participants reporting improved food security scores over the duration of the program. Notably, unlike many food prescription evaluations, FFRx used a validated tool from the Canadian Community Health Survey to assess food security, improving the comparability and validity of results.

Participants of FFRx significantly increased their self-reported consumption of fruit and ‘other’ vegetables (including cucumber, celery, corn, cabbage, and vegetable juice) over the 12 weeks from baseline to follow-up. Indeed, participants reported consuming almost double the servings of fruit and two additional servings of 'other' vegetables per week at follow-up in comparison to baseline; an improvement that can likely be attributed to participation in FFRx. However, participants reported no significant change in dark green vegetable consumption (e.g., broccoli, green beans, peas, green peppers, or dark leafy greens including lettuce or spinach). Such findings align with two recent studies tracking the dietary impacts of fresh fruit and vegetable prescriptions in the USA, which reported similar increases in fresh fruit and ‘other’ vegetable consumption among children and adult participants [33, 34]. Furthermore, results showed that FFRx program utilization may affect overall benefits. Specifically, participants who were frequent users (i.e., those who used ≥50% of their vouchers) reported a large and statistically significant improvement in consumption frequency of total fruit and total vegetables. Such findings provide further support for the potential benefits of FFRx and underscore the importance of establishing a program that addresses barriers to utilization. In particular, qualitative findings suggested that market-based dietary interventions should encourage use through frequent reminders, a welcoming and safe environment, and ensuring location and timing are convenient and physically accessible to participants. While this pilot study showed promising preliminary findings, longer and more robust studies incorporating a control group are needed to confirm that food prescriptions are a beneficial tool for promoting sustainable positive dietary changes.

We assessed self-reported health at baseline and follow-up using validated survey tools. Participants did not report a significant change to their health over the program; however, several participants mentioned in semi-structured interviews that the program improved their ability to manage chronic health conditions. It is likely that the short duration of FFRx limited the potential health impacts of the program. Furthermore, the implications of the COVID-19 pandemic on physical, mental, and social health are well-documented, especially among those experiencing existing vulnerabilities [35, 36]. Such effects likely confounded self-reported health outcomes at follow-up, thereby offsetting or occluding the potential benefits of FFRx. Previous studies have reported some significant, albeit small, changes to body mass index (BMI) and glycated hemoglobin (HbA1c) among food prescription program participants with cardiometabolic disorders, although additional evidence is needed to confirm such findings and fully understand the benefits and limitations of food prescriptions as an approach to promote clinical health and chronic disease management [33, 37, 38].

Participants reported additional benefits of FFRx beyond the food security, dietary, and health impacts. In semi-structured interviews, several respondents championed the social benefits of participation, as markets provided an opportunity for participants to socialize with staff and other customers. A recent systematic review identified social factors (including vendor-consumer relationships and social shopping with friends and family) as facilitators of farmers’ market patronage among low-income populations [39]. Our study provides further evidence for positive social environment as a facilitator of food market attendance and is the first to report such findings within the context of a targeted food prescription program. Similarly, involvement in FFRx increased participants’ utilization of other programs offered by the CHC from baseline to follow-up, which corresponds with findings of Marcinkevage and colleagues (2019), who reported that involvement in a food prescription program encouraged healthcare providers to refer food insecure patients to additional community services [40]. Results therefore indicate that using fresh food prescription can improve patient-provider communication to further accommodate patient needs. Based on these findings, future food prescription programs should ensure a welcoming, accessible, and social market environment and opportunities for extended interactions with healthcare practitioners to increase program utilization among participants.

Improving the affordability of fresh fruits and vegetables is a key component of fresh food prescription programs with food insecure participants. High prices for produce is often cited as a barries to healthy eating, especially for low-income households [30, 33, 40]. While no other studies have conducted a formal evaluation of voucher price acceptability, nor reported on voucher usability, several other fresh food prescription programs reported similar subsidies (e.g. US $7/person/household/week [32, 33] or US $40/month/participant [30]) and were effective at increasing the affordability of healthy eating. FFRx’s weekly CAD $10 per person per household was an appropriate amount for increasing healthy food affordability amongst most participants in this study. Transportation has been cited as an external cost that reduces food affordability [30], but such costs may be reduced by ensuring convenient locations of food retailers and markets (e.g., within healthcare centres similar to the CHCs) [28]. As yet, no analyses have evaluated the financial cost of food prescription programs against their health benefits. Future research should determine whether such interventions comprise an efficient use of financial and human resources to address food insecurity and reduce healthcare burdens across populations, sub-populations, and contexts.

Due to the COVID-19 pandemic, FFRx shifted to home delivery of prepared produce boxes, allowing us the opportunity to compare delivery models. Results suggest that most participants prefer interventions that allows for consumer choice, rather than prepared fresh food boxes. This finding was echoed by research conducted by Saxe-Custack and colleagues (2018) that compared the effectiveness of farmer's market vouchers to prepared bags of produce in a fresh food prescription intervention [28]. During the weeks following the shift to home delivery, participants’ consumption of fruits and vegetables was constrained by the contents of the produce boxes. Since produce boxes did not incorporate participants’ individual preferences, many individuals reported increased food waste and food sharing following the transition to this delivery model. Food sharing has not been described in previous evaluations of food prescription programs, and while this may benefit social relationships between participants and community members, it also may reduce consumption of fruits and vegetables by the participants themselves.

Food prescriptions, along with other social prescribing programs, offer a promising opportunity for healthcare providers to link patients with sources of support within their community in lieu of (or in addition to) pharmaceutical interventions [15–17]. Such programs align with recent calls for improved patient-centred healthcare that centralizes patient preferences, needs, and experiences by addressing underlying social determinants of health [41]. It should be noted that these supports are, at best, a stopgap measure and do not comprise a permanent solution to the root causes of food insecurity and poor health, which are often linked to poverty and systemic inequities [42]. Nevertheless, this study establishes a strong justification for further inquiry of the potential of fresh food prescriptions as a way to leverage patient-provider relationships to improve food security, dietary adequacy, food literacy, nutrition, and health. Targeted food prescriptions should be evaluated for cost effectiveness, acceptability, and impacts against existing community food provision models (e.g., food banks) to determine their relative benefits as models for community food assistance.

Our study identified a number of factors that contributed to participants’ positive experiences with FFRx, which should inform future iterations and adaptations of fresh food prescription programs. First, corresponding with previous evidence on community-based food interventions, a safe and welcoming social environment contributed to the acceptability and accessibility of FFRx and alleviated concerns of stigma among participants [30, 33]. Participants also appreciated the discreetness of vouchers to minimize possible stigmatization at community markets. Second, FFRx participants noted that extended interactions with participating healthcare practitioners were beneficial for alleviating initial uncertainties regarding the program, aligning with evaluations of food prescription programs elsewhere [29, 30]. Third, any fruit and vegetable prescription program should offer a wide variety of high quality, acceptable, and culturally appropriate produce. Fourth, food prescriptions should be of sufficient monetary value to ensure participants can obtain adequate amounts of healthy food and should be structured in a way that provides freedom of choice to participants.

This study had several limitations. Survey tools often used questions that were categorically arranged (e.g., yes-no or true-false) or provided a limited range of responses (e.g., five-point Likert scales), leading to measures scaled in discrete units that may lack the sensitivity to detect small changes over a short intervention period [43, 44]. We did not collect market data on voucher usage, so we were unable to validate self-reported voucher utilization or examine associations between program fidelity and primary outcomes. The study examined self-reported health outcomes but did not collect anthropometric or clinical data on participants’ cardiometabolic health or micronutrient deficiencies, limiting our capacity to determine the clinical health impacts of the intervention. Importantly, no control group was used in this study, and the limitations of one-group pre-post measures study designs are well described, including lack of randomization and threats to external validity [45]. The response rate was less than 65%, and loss to follow-up may have introduced bias; however, retained participants were not notably different from participants lost to follow-up in regard to any outcomes evaluated by baseline surveys (e.g., food security, self-reported health, and fruit and vegetable consumption; results not shown). Finally, collecting data on other food groups (in addition to fruits and vegetables) would provide a better assessment of the program’s impact on overall diet.

## Conclusion

This article described an evaluation of a fresh food prescription pilot program based at two locations of the Guelph CHC in Guelph, Ontario. The FFRx project showed that prescribing fresh fruit and vegetables for food insecure patients with a diet-related illness has the potential to address food security and promote beneficial dietary changes and social connectivity. While this program may not offer a long-term solution to food security or nutritional health issues, it offers healthcare providers a useful tool to reduce barriers to healthy eating by improving the availability, accessibility, affordability, acceptability, and accommodation of healthy foods. During interviews, participants perceived the program as beneficial to their diet and nutritional health, household food security, and social connectivity. Key aspects contributing to the success of the program included its social environment, integrated approach with health providers and the SEED staff, flexibility in how the vouchers cards can be used, accessibility of markets, and the quality and price of the produce at markets. More evidence is needed to determine if food prescription programs have physical and/or mental health benefits using rigorous study designs incorporating a control group. Food prescription interventions have potential to leverage recent calls for patient-centred care to promote and incentivize healthy dietary choices to improve nutrition, food security, health, and quality of life.

## Data Availability

The data that support the findings of this study are available from the Guelph Community Health Centre but restrictions apply to the availability of these data, which were used under license for the current study, and so are not publicly available. Data are however available from the authors upon reasonable request and with permission of the Guelph Community Health Centre.

## Abbreviations

BMI: Body mass index
CCHS: Canadian Community Household Survey
CHC: Community Health Centre
COVID-19: Coronavirus Disease 2019
FFRx: Fresh food prescription pilot program
HbA1c: Glycated hemoglobin
ODPS: Ontario Disability Support Program
USA: United States of America
USD: United States Dollars
